# Nanofluid based on self-nanoencapsulated metal/metal alloys phase change materials with tuneable crystallisation temperature

**DOI:** 10.1038/s41598-017-17841-w

**Published:** 2017-12-14

**Authors:** Nuria Navarrete, Alexandra Gimeno-Furio, Rosa Mondragon, Leonor Hernandez, Luis Cabedo, Eloisa Cordoncillo, J. Enrique Julia

**Affiliations:** 1 Universitat Jaume I. Departamento de Ingenieria Mecanica y Construccion, Castellon de la Plana, 12071 Spain; 2 Universitat Jaume I. Polymers and Advanced Materials Group, Castellon de la Plana, 12071 Spain; 3 Universitat Jaume I. Departamento de Química Inorganica y Organica, Castellon de la Plana, 12071 Spain

## Abstract

Nanofluids using nanoencapsulated Phase Change Materials (nePCM) allow increments in both the thermal conductivity and heat capacity of the base fluid. Incremented heat capacity is produced by the melting enthalpy of the nanoparticles core. In this work two important advances in this nanofluid type are proposed and experimentally tested. It is firstly shown that metal and metal alloy nanoparticles can be used as self-encapsulated nePCM using the metal oxide layer that forms naturally in most commercial synthesis processes as encapsulation. In line with this, Sn/SnOx nanoparticles morphology, size and thermal properties were studied by testing the suitability and performance of encapsulation at high temperatures and thermal cycling using a commercial thermal oil (Therminol 66) as the base fluid. Secondly, a mechanism to control the supercooling effect of this nePCM type based on non-eutectic alloys was developed.

## Introduction

The thermal properties of Heat Transfer Fluids (HTF) play a key role in the efficiency of thermal-based industrial processes such as electricity generation, oil and gas processing, chemical industry, and manufacturing processes, among others. From the environmental perspective, improving the efficiency of thermal systems is crucial since 90% of the world’s energy budget centres on heat conversion, transmission and storage. Minor improvements in the efficiency of thermal systems can significantly reduce CO_2_ emissions^1,2^.

Thermal oils, glycols and molten salts are used as HTF in medium- and high-temperature applications because they present good stability under high-temperature conditions. However, the thermal properties of these HTFs are quite poor, with thermal conductivity (k) and specific heat capacity (c_p_) values below 0.4 W/m·K and 2.5 J/g·K, respectively. Nanofluids are defined as engineered colloidal suspensions of nanoparticles in a base fluid. Nanofluids allow a solid to be introduced into a liquid, transferring to some extent the solid thermal properties to the liquid and keeping, also to some extent, its liquid transport properties. Therefore, nanofluids present an effective route to improve the thermal properties of HTF^3,4^.

The earliest nanofluid works were related to thermal conductivity enhancement using water as the base fluid^5–7^. Afterwards, nanofluid-related research topics were extended and nanofluid viscosity and specific heat were involved^8^. Nanofluid thermal and rheological properties depend on the base fluid but, broadly speaking, it is possible to observe for a certain base fluid, nanoparticle morphology and a given nanoparticle concentration value, a more marked increment in viscosity than that in thermal conductivity. In non-ionic fluids, a slight drop in the specific heat of the nanofluid is also expected^9^. A few industrial applications exist in which nanofluids with such physical properties can be found of interest^10^.

From 2010, new nanofluid approaches that use additional solid particle properties have been investigated. One of them is to use nano-encapsulated Phase Change Materials (nePCM) as the solid phase. In this approach, nanoparticles have a solid-liquid PCM core and a high melting temperature shell that keeps the PCM confined when in the liquid phase. Use of nePCM allows both the thermal conductivity and heat capacity of the base fluid to increase by the latent heat contribution of nePCM’s cores. Metal and metal alloy nanoparticles can be used as nePCM’s cores suitable for medium- and high-temperature applications. Metal and metal alloy nanoparticle melting and crystallisation temperatures and enthalpies have been studied in detail in recent years. In most cases, encapsulation is obtained by inserting nanoparticles into a high melting temperature matrix^11–17^, or by growing a silica^18–23^ or trioctyl phosphine oxide TOPO^24^ shell around them. Different metal cores have been investigated: indium^11,16,18,19^, bismuth^11–13,16,17^, tin^11,16,20–23^, cadmium^11^, lead^11,15^, zinc^24^, and their alloys^14,17,25^. Generally speaking, if metal cores are lower than 50 nm, lowering melting temperature and melting and crystallisation enthalpy values than those measured in the bulk material are observed due to the substantial contribution of the metal atoms near the shell^26,27^. In addition, lower crystallisation temperature values, defined as supercooling, are always found for this small nePCM. The purity of the materials in the core and their small size prevent heterogeneous crystallisation as there are no nucleation spots inside the nuclei. Thus, homogeneous nucleation takes place for crystallisation at a lower temperature. The nePCM supercooling temperature depends on the nucleus’ material and size.

One important parameter in shell-type nePCM is the encapsulation ratio, which is defined as the ratio between the phase change enthalpy per unit of mass of nePCM and that of the PCM bulk material. Encapsulation ratios below 1 are due to both the mass contribution of the nePCM shell, which does not melt within the working temperature range of nePCM, and the reduced enthalpy due to size effects (only for nePCM whose nuclei are smaller than 50 nm). In order to maximise the nePCM latent heat contribution, the encapsulation ratio should be as high as possible, assuring shell mechanical integrity.

Regarding the shells that surround nePCM’s cores, silica and TOPO shells have been previously used in nePCM-based nanofluids, where a wide range of base fluids, such as methanol^18^, molten salt^21,24^, poly-alpha-olefin (PAO)^19^ and commercial thermal oil (Therminol 66)^22^, have been used.

NePCM offer considerable advantages over conventional nanoparticles, such as major and better controlled heat capacity increments. However, two main drawbacks limit their application in nanofluids. On one hand, complex chemical synthesis processes are needed to obtain shell-type nePCM, which involve at least four chemical processes: one to produce metal nanoparticles, a second one to grow a polymeric template around them, a third one to grow the silica or TOPO shell on the template, and a final one to eliminate the polymeric template. On the other hand during the heat transfer process, nePCM’s cores should be melted and crystallised. Therefore, the difference between HTF charge and discharge temperatures should at least be the same as between the melting and supercooled crystallisation. This way, supercooling limits the impact of the nePCM latent heat contribution to the heat capacity of the base fluid, as the energy absorbed in the charging stage of the process will be released at a lower temperature in the discharge.

In this work, two new approaches to overcome the drawbacks of nePCM are proposed and experimentally checked. It is firstly shown that a metal oxide shell is produced during the metal nanoparticle fabrication process by standard commercial methods, which can be used as self-encapsulation for static and dynamic conditions to avoid complex chemical processes to create shell-type nePCM. It is secondly shown that non-eutectic metal alloy cores can eliminate the supercooling effect by using solid-phase remainders as nucleation spots during the crystallisation process. This latter approach, along with the proper alloy composition selection and a known value of the maximum working temperature of the HTF, enables a form of thermal management of the nePCM inside HTF with a tunable discharge temperature.

## Results and Discussion

### Self-nanoencapsulation by a metal oxide layer

**Figure 1:**
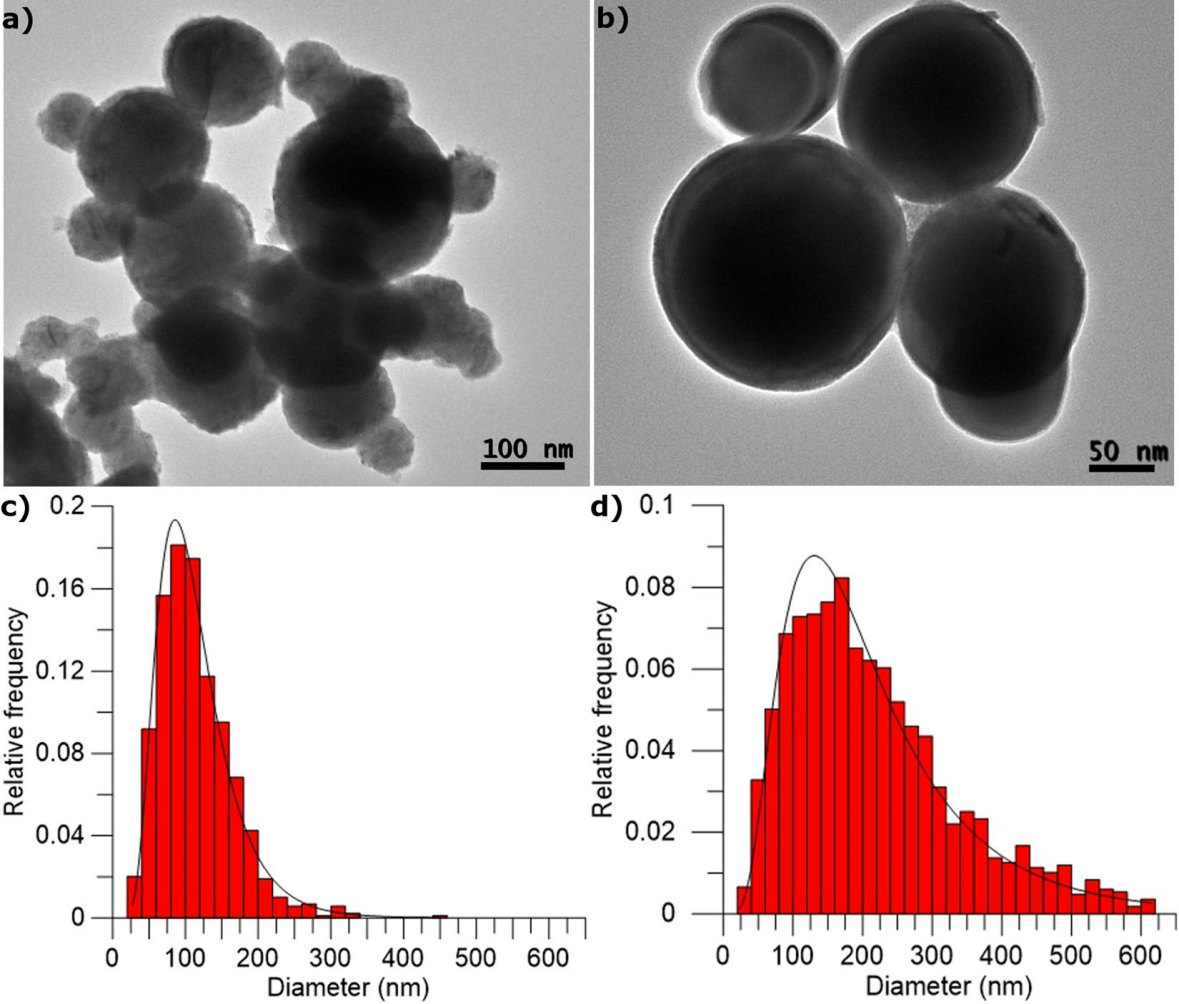
Comparison of the properties of the two samples, Sn of nominal size 60–80 nm and <300 nm. TEM image of Sn (**a**) 60–80 nm and (**b**) < 300 nm. Size distribution of Sn (**c**) 60–80 nm and (**d**) < 300 nm.

Nanoparticle size distribution was evaluated for two commercial Sn nanoparticle types, one with a nominal size of 60–80 nm and the other with one of <300 nm, as seen in Fig. 1. Depicted size distribution shows that the mean diameter of nanoparticles is larger than the value provided by the supplier, especially for the Sn 60–80 nm sample. Both samples’ particle size follows a lognormal distribution, centred on 103 nm for commercial 60–80 nm nanoparticles, and on 184 nm for commercial <300 nm ones. The presence of the shell covering the metallic nucleus is seen as a slightly paler layer in both images (Fig. 1(a and b)). The XRD performed for both samples (spectra are shown in Supplementary Figure S1) shows only crystalline pure Sn from the nePCM core, which proves the initially amorphous nature of these metal oxide shells.

The relationship of shell thickness with the nePCM particle diameter size was studied. Both measurements were calculated from TEM images for more than 175 particles. Only the shell thickness in the nePCM particles under 200 nm was measured as bigger particles were too dense and casted a shadow that did not allow them to be identified in the TEM images. Regardless of nePCM size however, the shell thickness values still fell around 9.78 nm (see Supplementary Figure S2).

Therefore from the TEM images analysis, nePCM shell thickness was found to be 9.78 ± 2.5 nm, regardless of particle size.

**Figure 2:**
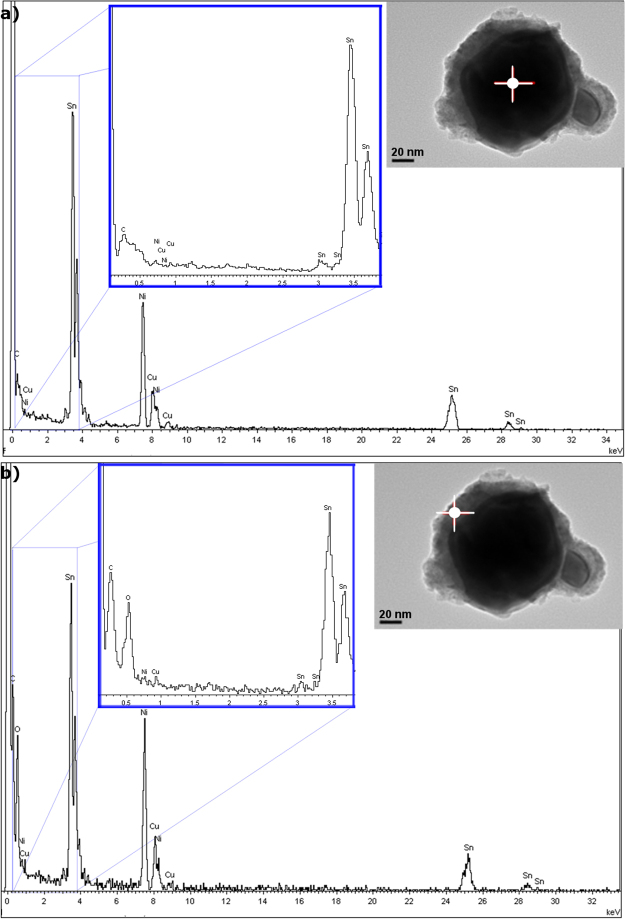
EDS analysis of the Sn nePCM: (**a**) of the core and (**b**) of the encapsulating shell (spot size: 10 nm).

In Fig. 2 it can be observed how the encapsulating layer in Sn nanoparticles is composed of Sn oxide, as their compositional analysis, performed with an EDS spot size of 10 nm, showed a higher content in oxygen than that of the nucleus.

**Figure 3:**
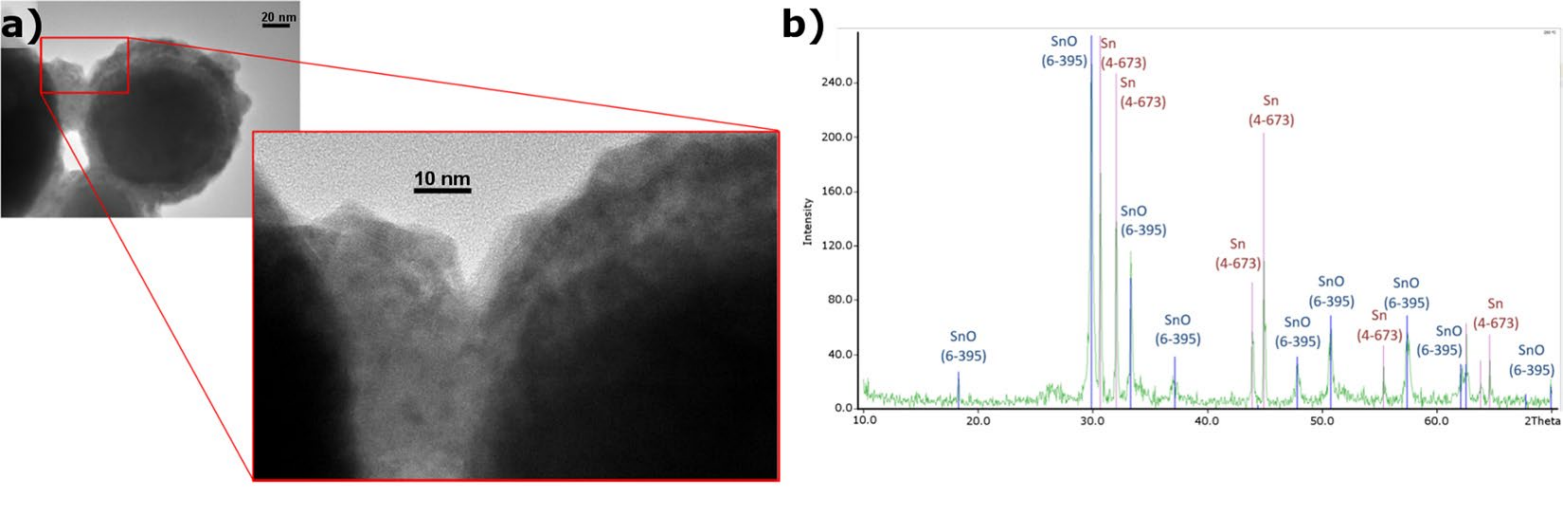
Encapsulating layer characterisation. (**a**) Detail of the TEM image of the oxide shell where a crystalline structure can be seen. (**b**) XRD analysis of the sample after exposure to high temperature (280 °C, 3 h).

Sn nanoparticles were exposed to high temperature (280 °C) for 3 hours in a nitrogen atmosphere to evaluate their response. Figure 3 shows how the initially amorphous oxide shell became partially crystalline when exposed to high temperature. Figure 3(a) depicts a TEM image where crystalline planes can be seen. Figure 3(b) shows the SnO peaks that corresponded to the nanoparticle shell which appeared in the XRD analysis.

### Nanoencapsulation stability after thermal cycling and maximum working temperature

**Figure 4:**
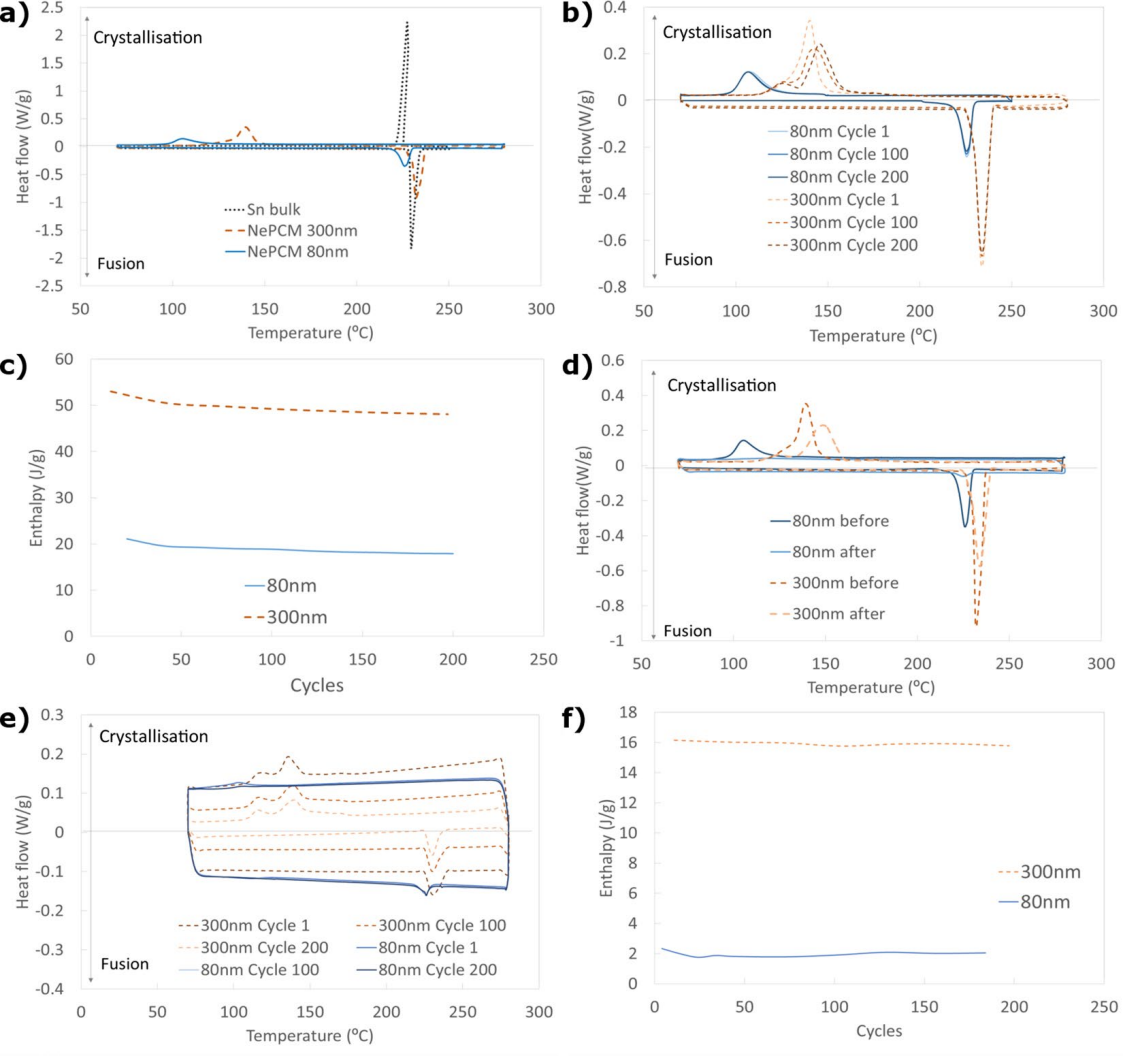
Differential Scanning Calorimetry (DSC) analysis of nanoencapsulation suitability and integrity. (**a**) Comparison of the properties of both nePCM samples with bulk Sn. Evolution of the (**b**) behaviour and (**c**) enthalpy of both samples (in powder) with thermal cycling. (**d**) Behaviour of samples before and after breakage. Evolution of the (**e**) behaviour and (**f**) enthalpy of both nanofluids composed of the Sn nePCM and TH66 with thermal cycling.

The phase-change characteristics of both nePCM and a piece of approximately 40 mg of bulk Sn were studied by thermal cycling. The comparison in Fig. 4(a) shows that the difference between both nePCMs and the bulk Sn melting points is almost negligible, with 229.5 °C for the bulk, 225.5 °C for the Sn 60–80 nm, and 232.1 °C for the Sn <300 nm. These results agree with the expected behaviour as the different melting temperatures depend on the size of nePCM^26^. However, a remarkable difference in their crystallisation temperatures was noted as the phenomenon known as supercooling appeared. This meant that the materials in the nucleus crystallised at much lower temperatures than those they melted at due to a small particle size and the absence of nucleation points. It was observed that the difference between the melting and crystallisation temperatures was only 2.3 °C for the bulk Sn, but 118.9 °C and 90.3 °C for the 60–80 nm and <300 nm Sn nanoparticles, respectively. Hence supercooling temperature was lower for the smallest particles, but was noticeable for both studied types^28^.

It was also observed that phase-change enthalpies were considerably greater for the larger nePCM, with values of 48 J/g for the Sn < 300 nm sample compared to that of 18 J/g for the Sn 60–80 nm sample. This was expected as they presented a higher encapsulation ratio, which meant that a bigger part of the total mass of the sample was pure Sn nuclei, which melted and crystallised. By taking into account the enthalpy values obtained for both samples and for the Sn in bulk form (a phase-change enthalpy of 60 J/g), 30% encapsulation ratios for the Sn 60–80 nm and 80% ones for the Sn < 300 nm were obtained.

This trend was also observed when the encapsulation ratios for each sample were mathematically estimated, with values of 48 ± 8% of total encapsulation ratio for the Sn of nominal size 60–80 nm, and 68 ± 6% for the Sn of nominal size <300 nm. In this case, the total encapsulation ratio refers to the relation between the volume of nuclei and the total volume of nanoparticles, including a correction factor for the values of particles smaller than 120 nm in diameter according to the decrease they present in their phase-change enthalpies^26^

Figure 4(b and c) illustrate the evolution of the nePCM in a powder form when thermal cycles are applied. The enthalpy values are slightly lower in the first 50 cycles, around 15% in Sn 60–80 nm and 9% in Sn < 300 nm, but become stable later. It is also noticed that while commercial 60–80 nm nePCM behaviour remains constant, the crystallising behaviour of the <300 nm particles varies from a single peak to two different ones. This is assumedly due to changes in the sample’s nuclei diameter distribution.

Similar data are obtained from the analysis of the nePCM nanofluids, which consisted in a 30% mass concentration of the nePCM in thermal oil Therminol 66, shown in Fig. 4(e and f). It can be observed that the stability in the enthalpy values is greater for the nanofluid than for the nePCM in a powder form, and remains practically constant for the <300 nm nanofluid sample.

Encapsulating shell resistance was tested and is shown in Fig. 4(d), where the phase-change enthalpies (corresponding to the peak area in each of the curves) clearly reduced after the breakage thermal cycle up to 400 °C. This drop in enthalpy values was 87% in the Sn 60–80 nm sample and 13% in the Sn < 300 nm one. This behaviour was expected as the covering shell breakage of some nePCM and their following exposure to air caused their nuclei to oxidise. Hence the pure material volume available to the change phase reduced.

**Figure 5:**
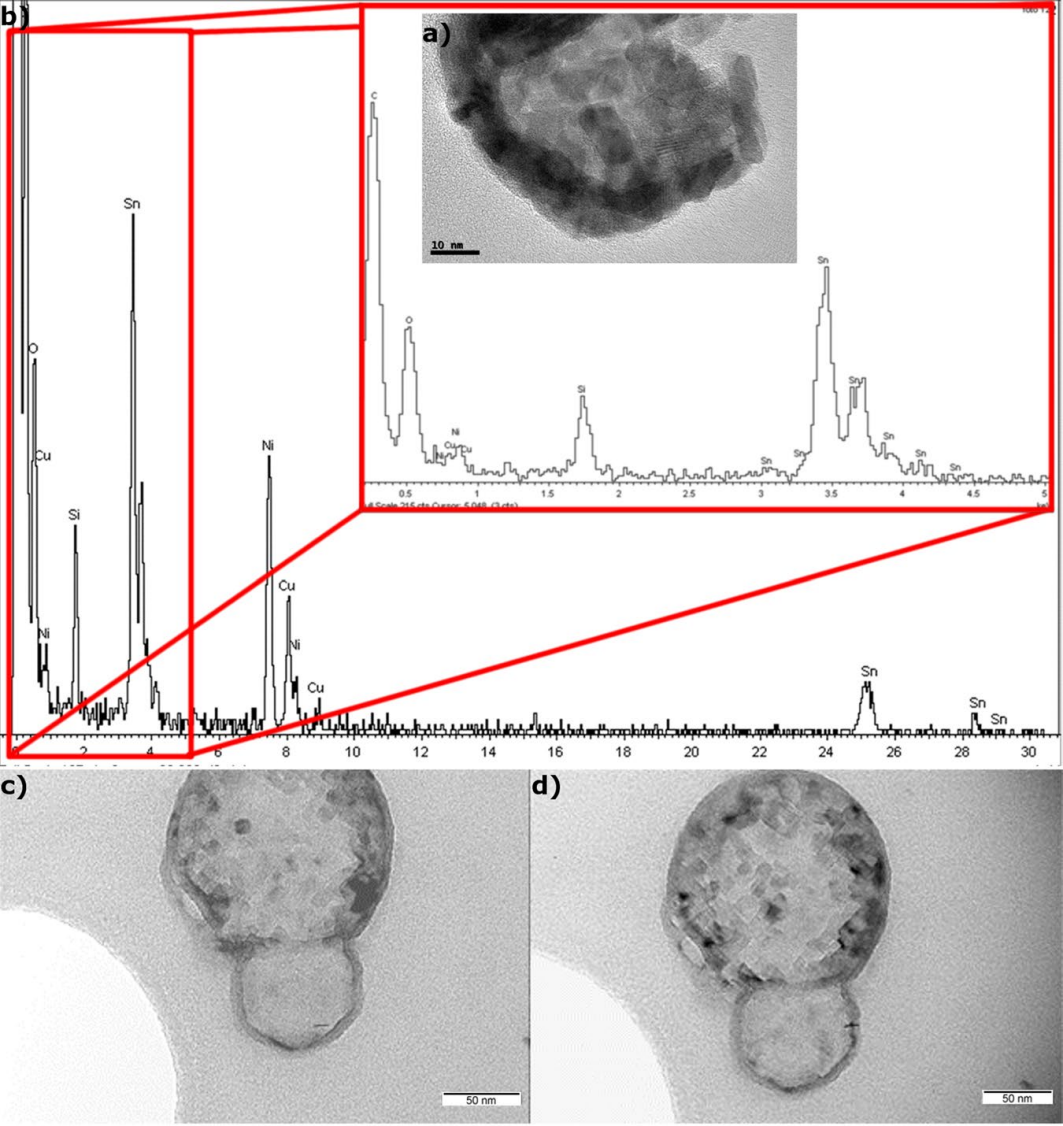
Integrity and breaking point of encapsulation. (**a**) TEM image and (**b**) EDS analysis of broken encapsulation after surpassing the breakage temperature, where the pure metal of the nucleus became SnO. High-temperature TEM images of Sn/SnO nePCM at (**c**) 70 °C and d) 300 °C.

The breakage and oxidation of the nucleus when exposed to temperatures up to 400 °C is observed in Fig. 5(a), where the crystalline nature of the oxide can be seen in the former particle nucleus, and the higher content in oxide is shown in the EDS analysis in Fig. 5(b).

Nevertheless, encapsulation integrity was proved for 300 °C, as it can be seen in the HT-TEM images in Fig. 5(c and d), where the oxide that covers the shell remains unaltered at this temperature and no breakage is observed.

### Performance comparison between base fluid and nePCM nanofluid

**Figure 6:**
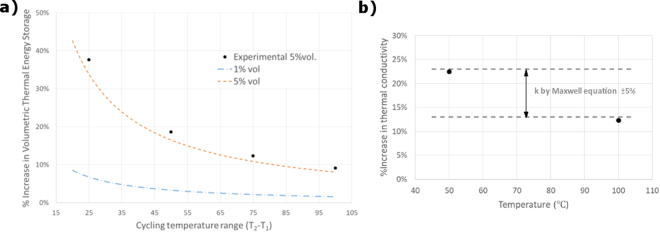
Comparison of nanofluid and base fluid performance. (**a**) Increase in volumetric thermal energy storage capacity for 1% and 5% vol. loading of Sn < 300 nm nePCM in TH66 as a function of the cycling temperature range. Dotted lines correspond to predictions. Black points correspond to experimental measurements for the 5% nePCM loading. (**b**) Experimental values of thermal conductivity for the base fluid and nanofluid with 5%vol. loading, and correlation with the values predicted by Maxwell equation.

A comparison of behaviour between the base fluid and nanofluid with Sn < 300 nm was made in order to prove the Thermal Energy Storage (TES) capacity and thermal conductivity increase of the latter. TES capacity is taken as the sum of the sensible heat and latent heat. In Fig. 6(a) it can be observed that there is an improvement in the performance of the nanofluid. This improvement depends on the cycling temperature range, since the latent heat enhancement by the addition of nePCMs has a bigger effect for smaller temperature increments, and as ΔT increases, the specific heat (sensible heat) has a stronger contribution than the phase-change enthalpy of the particles in the TES capacity.

Figure 6(b) shows the experimental values of thermal conductivity improvement obtained for 50 °C and 100 °C, and their agreement with the predicted values by using the Maxwell equation^3^.

### NePCM supercooling control by using non-eutectic metal alloys

As previously explained, supercooling occurs in nePCM and results in crystallisation taking place at lower temperatures than melting. Although this feature can be interesting for thermal regulation applications, one way of avoiding supercooling by using non-eutectic metal alloys was investigated.

**Figure 7:**
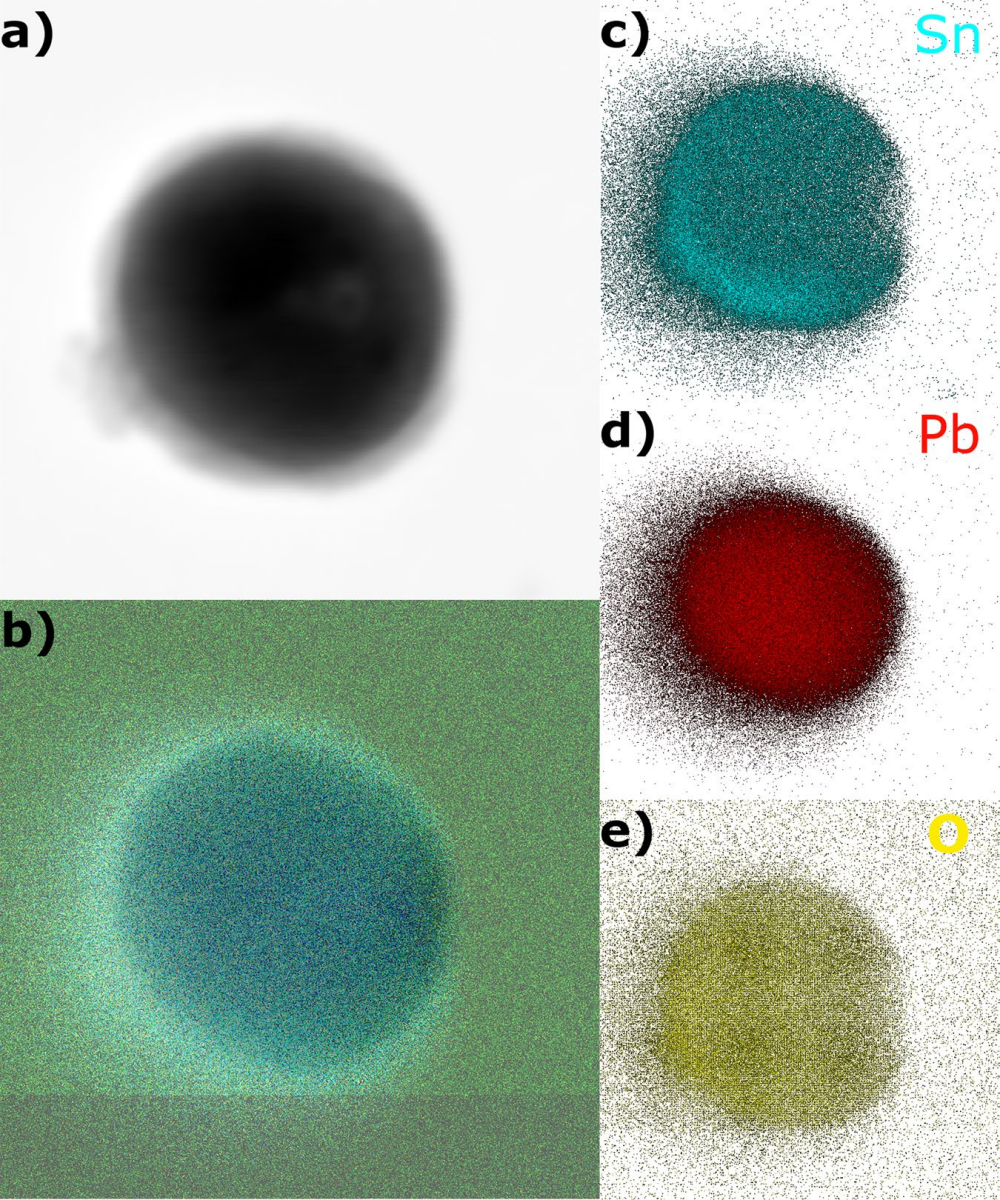
EELS image of a SnPb alloy nanoparticles where the composition is observed: (**a**,**b**) general view of the encapsulated nanoparticle. Composition of (**c**) Sn, (**d**) Pb and (**e**) O.

In-house metal alloy nanoparticles based on Sn and Pb were synthesised. Their composition is shown in the EELS analysis in Fig. 7. Here, it can be observed that the nucleus contains the Sn/Pb alloy, whereas the oxide that forms the encapsulating shell is mainly Sn oxide.

**Figure 8:**
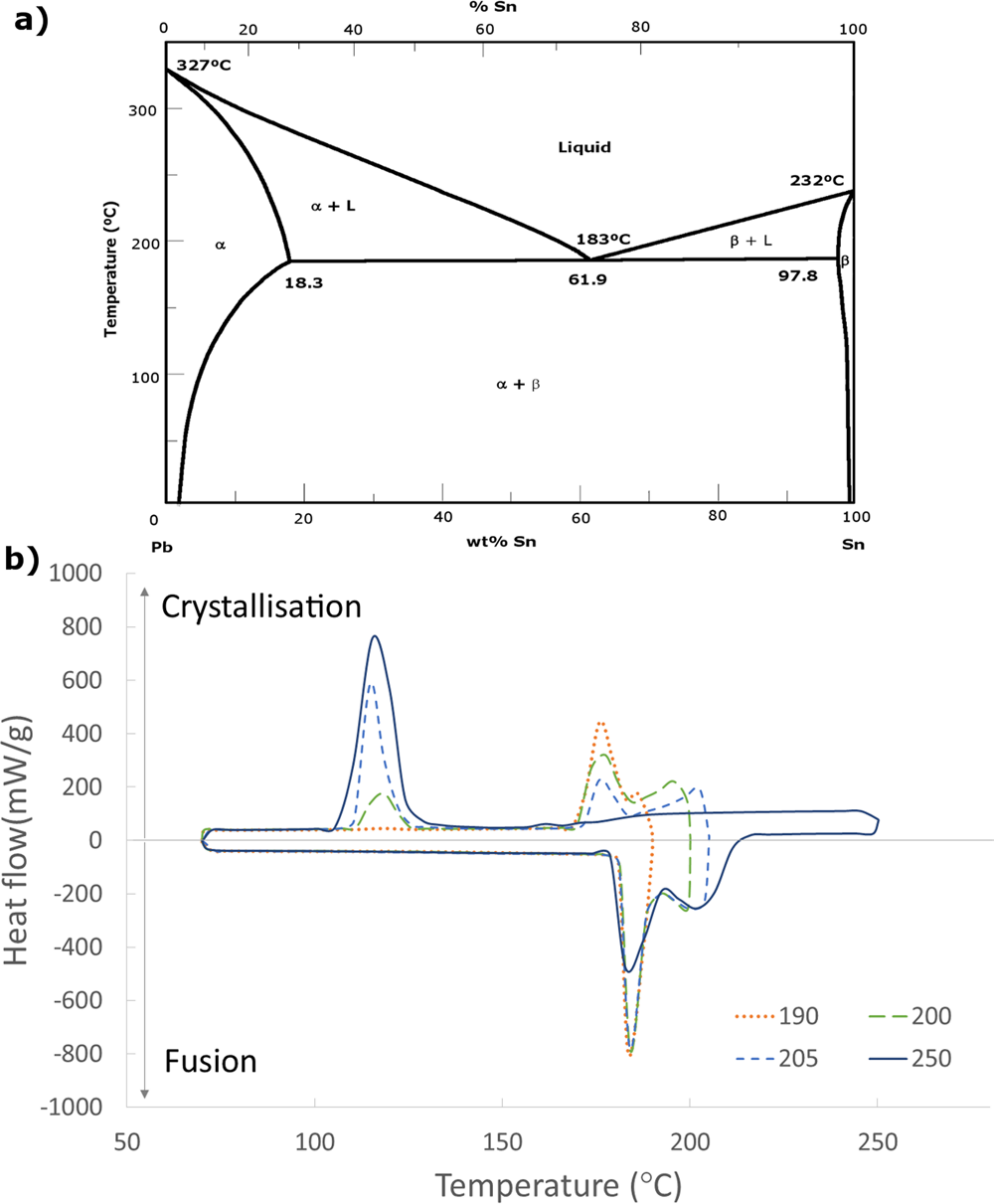
(**a**) Phase diagram of the Sn/Pb alloy and (**b**) DSC analysis of the Sn/Pb alloy’s thermal behaviour depending on the maximum temperature reached (between 190 °C and 250 °C).

According to the phase diagram in Fig. 8(a), for the non-eutectic alloys there is a range of temperatures within which a solid and a liquid phase co-exist. The remainders of this solid phase act as nucleation points for heterogeneous crystallisation, as opposed to the homogeneous nucleation that takes place in eutectic alloys or non-eutectic alloys when the whole nucleus is melted.

The alloy nePCM’s behaviour is seen in Fig. 8(b), along with thermal cycles with different maximum temperatures (between 190 °C and 250 °C). It can be noted that when nePCM’s nuclei do not completely melt crystallisation takes place at two different temperatures, first at a similar temperature to that of melting, and then at that which corresponds to the supercooling phenomenon.

It is also observed that the total crystallisation ratio which takes place at one temperature or another varies depending on how many of the nuclei melt. Thus, for higher temperature cycles, in which nuclei fusion is almost complete, most of the crystallisation takes place at the supercooling temperature, and only a small part occurs at a closer temperature to the melting one. As this trend is gradually inverted as fusion reduces, the cycles that reach lower temperatures show almost complete nuclei crystallisation at a similar temperature to the melting one, which avoids supercooling.

This behaviour is observed and quantified in Table 1, where the data of fusion and crystallisation enthalpies are shown. This table also shows the variation between both crystallisation peaks for different maximum cycle temperatures within the 190–250 °C range.

**Table 1:** Summary of the melting and crystallising enthalpies of the Sn/Pb alloy depending on the maximum temperature reached (between 190 °C and 250 °C).

Max. cycle T (°C)	T_m_ (°C)	ΔH_fusion_ (J/g)	T_c1_ (°C)	ΔH_cryst1_ (J/g)	T_c2_ (°C)	ΔH_cryst2_ (J/g)	ΔH_cryst1_/ΔH_total_ (%)	ΔH_cryst2_/ΔH_total_ (%)
190.00	184.14	27.48	175.64	27.16	—	—	100.00	0.00
195.00	184.30	28.17	175.48	28.37	120.22	0.77	97.36	2.64
200.00	184.30	33.70	175.74	30.52	117.00	6.44	82.58	17.42
202.50	184.30	37.48	175.64	22.81	115.41	14.86	60.55	39.45
205.00	184.31	39.54	175.74	22.24	115.20	21.01	51.42	48.58
210.00	184.48	40.70	173.76	0.58	115.37	42.25	1.35	98.65
250.00	184.48	44.62	—	—	114.85	44.23	0.00	100.00

Here it is also shown the percentage of the total crystallisation enthalpy that takes place during the first crystallisation (ΔH_cryst1_), with a temperature difference of around 9 °C with respect to the melting, and the second (ΔH_cryst2_) that presents a temperature difference of 69 °C (between the melting and supercooled crystallisation). T_m_, T_c1_ and T_c2_ refer to the melting temperature, and to the first and second crystallisation peaks, respectively.

## Conclusions

The suitability of a new nePCM type has been tested for its use in heat transfer nanofluids based on a synthetic thermal oil. The nanoparticles consist of a metallic nucleus covered by a metallic oxide shell that forms naturally during the fabrication process. This shell remains consistent throughout thermal cycling and prevents the material from the nucleus from leaking when in a melted state by resisting temperatures above the working temperature of the base fluid.

Addition of nePCM to a base fluid permits incremented heat capacity to be achieved due to the energy stored during the phase change, apart from the regular increment in thermal conductivity present in heat transfer nanofluids. The suitability of the metal oxide shell considerably lowers the costs to produce these nePCM compared with the techniques used to date.

A solution for the supercooling problem found in nePCM has also been studied. It consists in using non-eutectic metallic alloy nanoparticles in which the nucleus does not completely melt during the heating process. This leads to nucleation spots that allow crystallisation to happen at similar temperatures to melting ones.

## Methods

### NePCM preparation

Two types of nePCM were tested:

- Commercial Sn nanoparticles of nominal sizes 60–80 nm and <300 nm were purchased from US Research Nanomaterials, Inc. These nanoparticles were produced by the electrical wire explosion method^29^ and the electro-physical fumed^30^ method, respectively.
- In-house Sn/Pb alloy nanoparticles were synthesised using a modified polyole wet-chemical reduction process^23^. In a typical synthesis, 3.3 g of polyvinylpyrrolidone (PVP) (MW 40000, Sigma Aldrich) were dissolved in 55 mL of tetraethylene glycol (TEG) (99%, Sigma Aldrich) and heated to 140 °C. Then a SnCl_2_ solution (1.03 g in 12 mL of TEG) was added slowly to the reaction mixture. The PVP solution turned yellow-brown after adding the SnCl_2_ solution. Ten minutes later, a freshly prepared NaBH_4_ (99%, Sigma Aldrich) solution (1.3 g of NaBH_4_ in 15 mL of TEG) was added drop-wise to the reaction solution. Next a PbCl_2_ solution (0.115 g in 15 mL of TEG) was added. After 90 min, heat was removed and the solution was brought to room temperature. The reaction time and the amount of PVP allowed nanoparticle size distribution to be controlled. The entire synthesis was carried out with constant magnetic stirring with the protection of a N_2_ atmosphere. Sn/Pb nanoparticles were washed 3 times with ethanol, twice with distilled water and separated out by centrifuging.

### Nanofluid preparation

Nanofluids were prepared by adding nePCM to synthetic thermal oil HTF TH66 (Solutia, Inc.) at a 30% mass concentration. TH66 maximum operating temperature is 340 °C and 250 °C under closed circuit conditions and open conditions, respectively. The mixture was homogenised by sonication using a Sonopuls HD2200 (Bandelin) ultrasound probe in a 100% duty cycle for 5 minutes.

### NePCM and nanofluid characterisation

The size and morphology of the Sn and Sn/Pb nePCM were determined by direct observation under a Field Emission Transmission Electron Microscope (TEM, JEOL-JEM 2100) that operated at an accelerating voltage of 200 kV. The existence of the encapsulating oxide layer was also confirmed, and the analyses of the structure and qualitative differences in the composition of the core and shell were performed using the same microscope, which includes High-Resolution and EDS (Oxford INCA) analysis modules.

The quantitative determination of both the composition and the crystalline structure of the oxide layer were determined by X-Ray Diffraction (XRD) using a Bruker D8 X-ray diffractometer with Cu Kα radiation by studying the angles from 2θ = 10° to 2θ = 70° with a 0.02° step.

The thermal study to evaluate the melting and crystallisation temperatures and the associated enthalpies, existence of supercooling and thermal cycling to test their evolution through time and use was performed in a Differential Scanning Calorimeter (DSC2 Mettler Toledo). The Sn nePCM and nanofluid samples were subjected to 200 thermal cycles each, between 70 and 280 °C with 40 K/min heating and cooling rates, and 5-minute isotherms at maximum and minimum temperatures. Every 20 cycles, a more detailed analysis was done with the same temperatures and isotherms, but with heating and cooling rates of 5 K/min, to obtain the values of the melting and crystallisation temperatures and enthalpies.

The encapsulation ratio was calculated comparing the enthalpy/mass ratio obtained in the DSC analysis to that expected from the core bulk material, and also mathematically, according to the nuclei volume/ total particle volume ratio, where the shell thickness values were obtained from the TEM images.

The resistance of the encapsulating layer to high temperatures was tested by a High-Temperature Transmission Electron Microscope (HT-TEM, Philips CM 200) by obtaining images with the 70–300 °C range.

Encapsulation breakage was achieved using DSC, with a thermal cycle between 70 °C and 400 °C, and with 40 K/min heating and cooling rates and 5-minute isotherms at maximum and minimum temperatures.

The specific heat of the nanofluid was obtained using DSC dynamic method. Applying a known heating rate to the sample the specific heat can be calculated, since the heat flux measured by the DSC is directly related to specific heat, sample mass and heat rate applied.

Thermal conductivity was measured using the transient hot wire technique (KD2Pro, Decagon Devices) for 50 °C and 100 °C. The samples were put in a vial and submerged in a thermostatic bath to ensure temperature stability within the measurements.

The composition details of the Sn/Pb alloy nePCM were obtained with a Scanning Transmission Electron Microscope (STEM FEI TALOS F200X) and an EELS system, which allows the composition of sample atom by atom to be determined.

The data sets generated during, and/or analysed during, the current study are available from the corresponding author upon reasonable request.

## Electronic supplementary material

Supplementary information
