# Supplementary material for: Nanofluid based on self-nanoencapsulated metal/metal alloys phase change materials with tuneable crystallisation temperature
Source: Sci Rep. 2017 Dec 14;7:17580. doi: 10.1038/s41598-017-17841-w (PMC5730550; doi:10.1038/s41598-017-17841-w)
Supplement: Supplementary file 1 — Supplementary information [file 41598_2017_17841_MOESM1_ESM.pdf]

Nanofluid based on self-nanoencapsulated metal/metal alloys phase change materials with tuneable crystallisation temperature.

Nuria Navarrete[1], Alexandra Gimeno-Furio[1], Rosa Mondragon[1], Leonor Hernandez[1], Luis Cabedo[2], Eloisa Cordoncillo[3], J. Enrique Julia[1,*]

[1] Universitat Jaume I. Departamento de Ingenieria Mecanica y Construccion. Castellon de la Plana, 12071, Spain.

[2] Universitat Jaume I. Polymers and Advanced Materials Group. Castellon de la Plana, 12071, Spain.

[3] Universitat Jaume I. Departamento de Química Inorganica y Organica. Castellon de la Plana, 12071, Spain.

*enrique.julia@uji.es

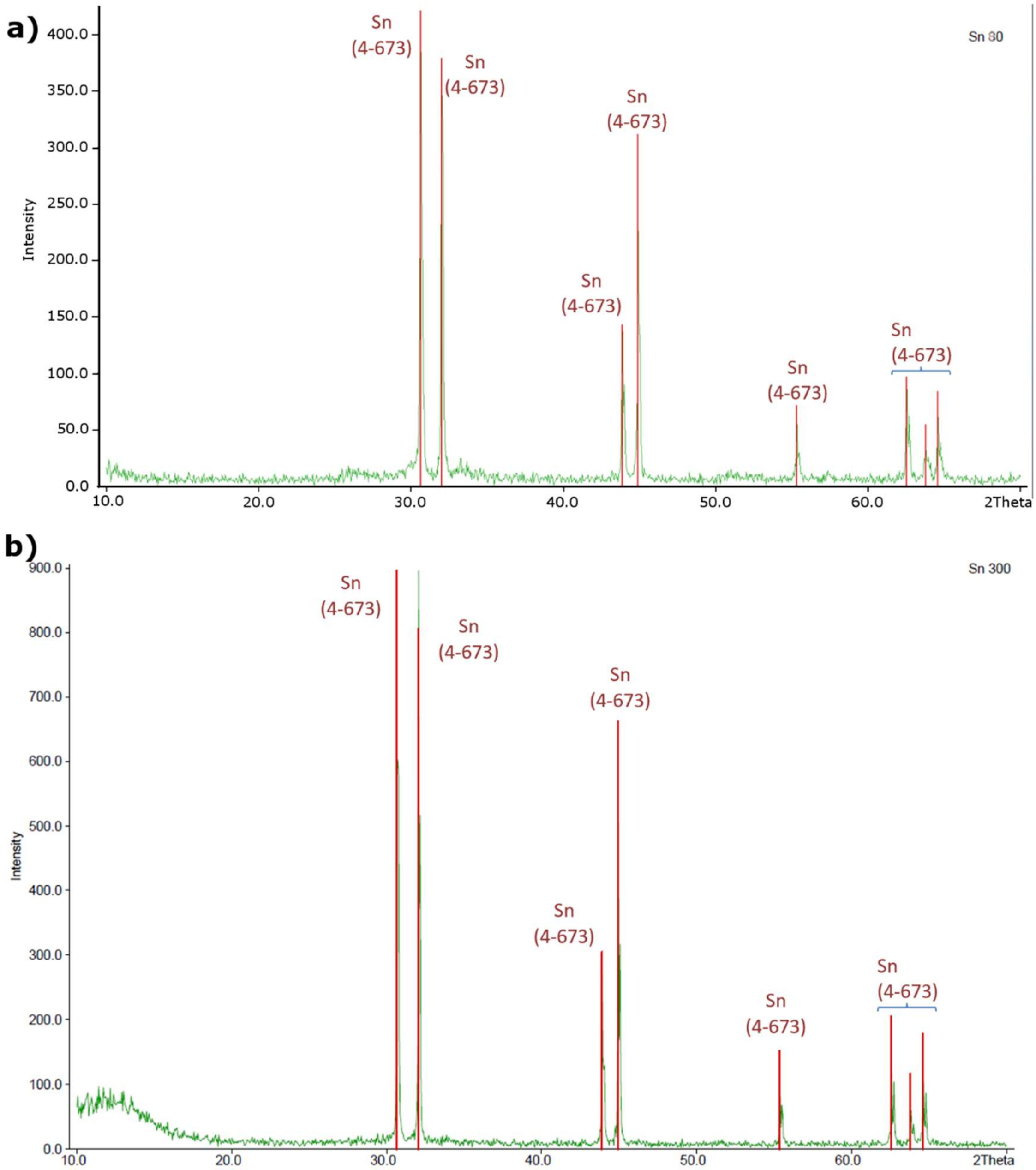

Figure S1. XRD analysis of both nePCM samples studied before thermal treatment: a) Sn of nominal size 60-80nm, and b) Sn of nominal size <300nm.

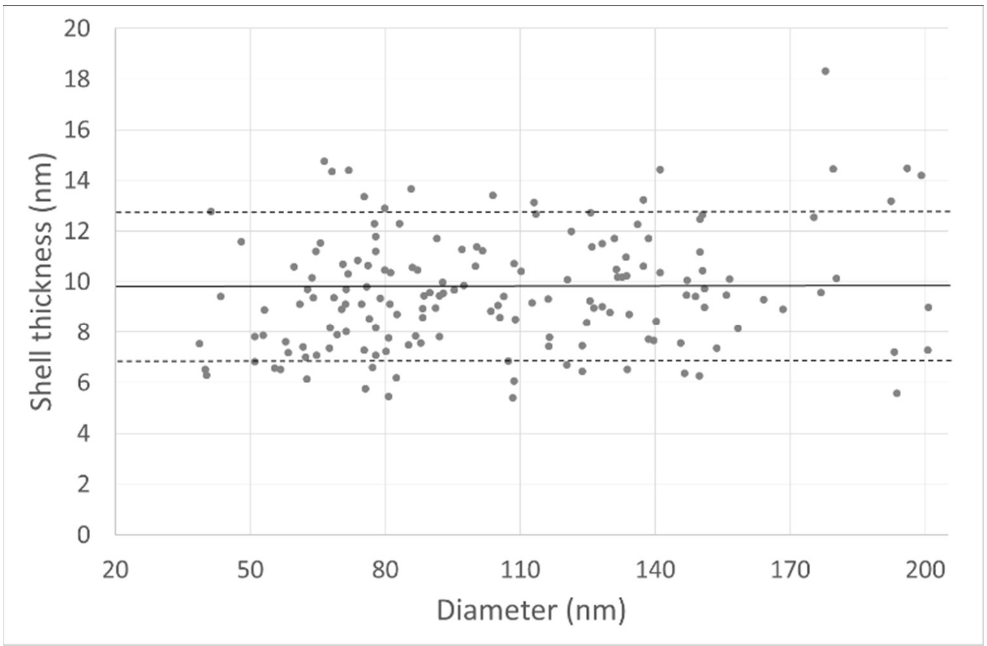

Figure S2. Shell thickness vs nePCM diameter distribution.

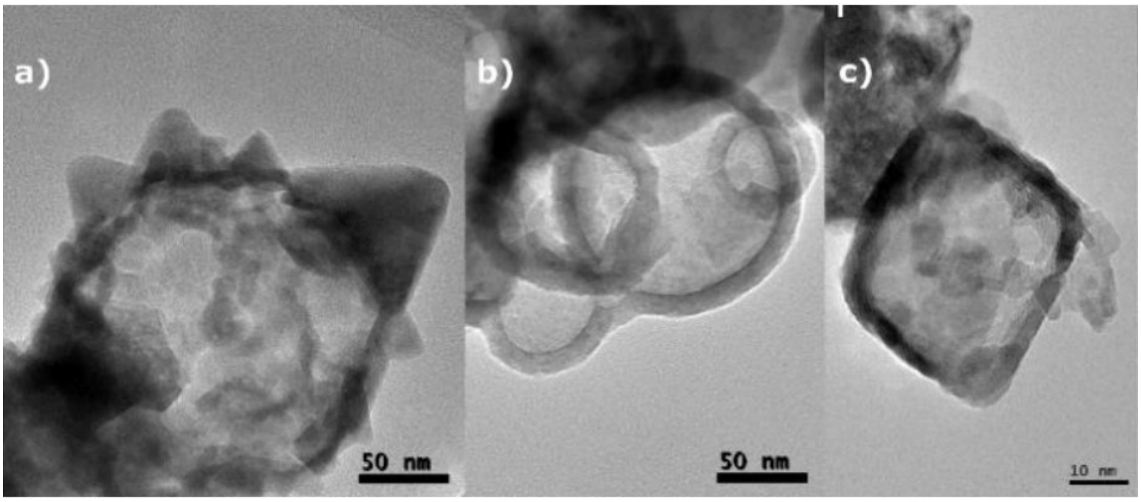

Figure S3. TEM images of other metallic nePCMs where the encapsulation by an oxide shell is observable: a) zinc, b) aluminium and c) indium.

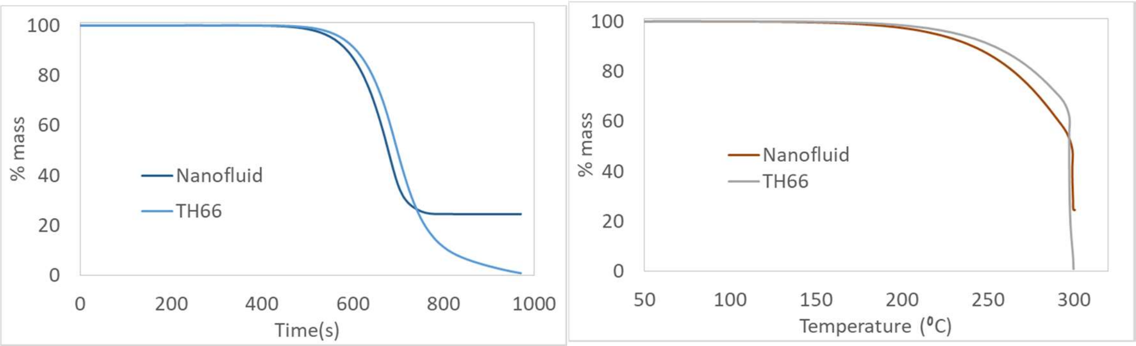

Figure S4. Thermogravimetric analysis of the base fluid (TH66) and nanofluid (TH66 + Sn<300nm nePCMs, 30%wt.) for temperatures ranging from 50 to 350ºC. A minimal difference between the base fluid and the nanofluid can be observed. Both samples show complete stability up to 180ºC, with no appreciable mass loss due to evaporation of the fluid. At 250ºC, when the cores of the nePCMs are completely melted, the 90% of the initial mass is still present for both samples. It has to be taken into account, that in real application of heat transfer fluids, the fluid is confined so auto-pressurization occurs, which was not the case in the thermogravimetric tests. This would lead to a wider range of temperatures for which the fluid remains completely stable.
